# Effects of infusion of different fluids during controlled hypotension on gastric intramucosal pH and postoperative gastroenterological function^☆^

**DOI:** 10.1016/S1674-8301(11)60025-0

**Published:** 2011-05

**Authors:** Guanglei Wang, Su Liu, Gongjian Liu

**Affiliations:** Department of Anesthesiology, the Affiliated Hospital of Xuzhou Medical College, Xuzhou, Jiangsu 221002, China.

**Keywords:** hemodilution, controlled hypotension, gastric mucosa

## Abstract

The present study was aimed to investigate the effects of infusion of different fluids combined with controlled hypotension on gastric intramucosal pH (pHi) and postoperative gastrointestinal function in patients undergoing hepatocarcinoma surgery. Forty-five patients (ASA II) scheduled for surgical resection of hepatocarcinoma undergoing controlled hypotension were randomly assigned to three groups and received infusion of 20 mL/kg Ringer's solution (R group), 6% HAES(H group) or 6% Voluven group (W group). Intragastric PgCO_2_, pHi, hematocrit and hemoglobin were measured. The significant decrease of pHi and increase of PgCO_2_ were produced at 1 and 2 h after controlled hypotension in the R group (*P* < 0.05 or *P* < 0.01). The time of bowel movement after operation was shorter in the W group than the R group. Meanwhile, we also did not find obvious difference in blood gas indexes among the three groups. The infusion of HAES and Voluven during controlled hypotension could improve gastrointestinal perfusion and accelerate the recovery of postoperative gastrointestinal function.

## INTRODUCTION

Hypervolemic hemodilution combined with controlled hypotension can lessen bleeding and save blood during surgery. It increases blood volume without increasing the cardiac load. It still remains unknown whether or not diluting blood under controlled hypotension will cause inadequate perfusion of vital organs and influence the microcirculation. Gastrotonometer can be used to evaluate localized blood flow of visceral vasculature. Compared with other conventional approaches, measurement of gastrointestinal mucosal pH using gastrotonometer is a considerably sensitive and specific method for estimation of microcirculatory blood flow and evaluation of the conditions of gastrointestinal blood perfusion and oxygenation[1]. The objective of this study is to investigate the effects of infusion of different liquids under controlled hypotension on gastric pH and gastrointestinal function after surgery using a gastrotonometer.

## MATERIALS AND METHODS

### General materials

After the study protocol was approved by the Institutional Review Board of local hospital and informed consent was obtained from the patients, we recruited forty five hepatic cancer patients [American Society of Anesthesiologists (ASA) II, male or female, age 25-62 years, and body weight 50-78 kg] undergoing elective surgery for this study. All the patients had normal hemoglobin levels, and normal hepatic and renal function. All the patients had no recent medication of nonsteroid anti-inflammatory drugs (NSAIDs) or steroid drugs and had no family history of hypertension, cardiovascular diseases, immunological or endocrinological diseases. Patients were randomly divided into three groups: 20 mL/kg Lactated Ringer's solution group (R group), 6% HAES steril group (H group) and 6% Voluven group (W group).

### Methods

All patients were fasted for 12 h for food and 6 h for drink prior to surgery. Midazolam (0.06 mg/kg) and scopolamine (0.3 mg) were intramuscularly injected at 30 min before anesthesia induction. Artery blood pressure, and mean artery blood pressure (MAP) were monitored through a catheter inserted in the radial artery in all patients. In addition, patients' heart rate and lead II ECG were continuously monitored. Central venous pressure was monitored through a catheter inserted into the intrajugular vein. The three groups of patients were replenished for the loss of the physiological requirements owing to fasting. Etomidate emulsions (0.3 mg/kg), fentanyl (6-8 µg/kg), and cisatracurium (0.2 mg/kg) were intravenously administered for general anesthesia induction in all patients. Propofol [5 mg/(kg·h)] and cisatracurium [3 µg/(kg·min)] were continuously infused through micro pumps, and 1.5MAC isoflurane was inhaled for general anesthesia maintenance. During 1 h after induction, three groups received infusion of different fluids at 20 mL/kg according to the host group followed by balanced solution for maintenance. Nitroglycerin was continuously given through the jugular vein initially at a dose of 0.3 µg/(kg·min) to keep MAP lower than the baseline level and CVP at 5-12 cmH2O. At the beginning of surgery, the dose of nitroglycerin was increased to induce 25% to 30% decrease of basic MAP[2],[3]. Controlled hypotension was terminated after tumor resection. All patients were given pure oxygen during the operation.

### Determination of gastric intramucosal CO_2_ pressure (PgCO_2_) and gastric intramucosal pH (pHi)

Gastric intubation was performed on patients with the gastrotonometer (TRIP-NGS) tubes. An automated air tonometry technique was used to measure PgCO_2_ using a catheter connected to a Tonocap monitor (Datex-Ohmeda Ltd., Hatfield, Herts, Finland). The catheter was inserted during anesthesia induction. Correct placement was confirmed by auscultating the epigastrium while 20 mL of air was insufflated through the nasogastric tube. The tonocap monitor automatically analyzed gastric gas samples for PgCO_2_ and simultaneously drew artery blood for gas analysis. The intramucosal CO_2_ pressure (PgCO_2_) and gastric intramucosal pH (pHi) were automatically calculated using the following formulas: pHi=pHa+lg(PaCO_2_/PgCO_2_) and Pg-aCO_2_=PgCO_2_-PaCO_2_[4]. All arterial blood gas measurements were obtained using the same blood gas analyser. The data before controlled hypotension was defined as the baseline value, and the value of artery blood gases and gastric mucosal pHi were recorded at 1 and 2 h after the onset of controlled hypotension, and 1 h after the termination of controlled hypotension. At some time point, blood was sampled for measuring hematocrit and hemoglobin (Hb).

### Statistical analysis

Data were expressed as mean±SD. Paired *t*-test was used to compare changes of pre-treatment and post-treatment, and one-way ANOVAs followed by a Newman-Keuls *post hoc* test were used to compare difference among three groups. *P* < 0.05 was considered as statistically significant. Statistical analyses of data were generated using the SPSS 13.0 package.

## RESULTS

All 45 patients in three groups completed the study protocol according to the anesthesia procedure, and all patients were induced by intravenous anesthesia, which was maintained by inhaled isoflurane successfully, and no patient was excluded.

### General information of patients in three groups

The general information for the three groups are presented in ***Table 1***. There were no significant differences among the three groups with regard to sex (male/female: the R group, 9/6; the H group, 8/7 and the W group, 7/8) (*P* > 0.05) and age [the R group, (46.2±3.5) years; the H group, (44.1±5.1) years; the W group, (46.3±4.8) years (*P* > 0.05)] and body weight [the R group, (61.1±7.8) kg; the H group, (59.4±8.2) kg; the W group, (62.3±8.6) kg (*P* > 0.05)]. The median duration of operation was similar among the three groups, (the R group, 180.4±48.6 min; the H group, (184.8±39.7) min; the W group, (178.5±42.7) min, (*P* > 0.05)].

**Table 1:** Comparison of general conditions of patients in hepatic cancer patients under controlled hypotension and receiving infusion of different fluids

Group	Sex (male/female)	Age (years)	Body weight (kg)	Operation time (min)
R group	9/6	46.2 ± 3.5	61.1 ± 7.8	180.4 ± 48.6
H group	8/7	44.1 ± 5.1	59.4 ± 8.2	184.8 ± 39.7
W group	7/8	46.3 ± 4.8	62.3 ± 8.6	178.5 ± 42.7

No significant difference in baseline characteristics was found among the three groups (*P* > 0.05). The H group: 6% HAES; the R group: 20 mL/kg Ringer's solution; the W group: 6% Voluven.

(mean±SD, *n* = 15)

### Changes of hematocrit at each time point in three groups

The hematocrits at each time point are displayed in ***Table 2***. There were no baseline differences between the groups. A progressive decline in the hematocrit was observed in the W group at 1 h after induction (31.5±1.5)% and during controlled hypotension (30.7±1.8)% comparing with the baseline value (43.9±5.7)% and that of the R group or H group at 1 h after induction [(39.5±4.2)% and (33.6±1.9)%, respectively], during controlled hypotension [(38.3±5.4)% and (32.2±3.9)%, respectively]. However, there is no difference in the hematocrit values between the R group and H group not only at 1 h after induction (*P* > 0.05) but also during controlled hypotension (***Table 2***).

**Table 2:** Changes of hematocrit at each time point in hepatic cancer patients under controlled hypotension and receiving infusion of different fluids

Group	Basic date	1 h after induction	During controlled hypotension
R group	44.1 ± 5.8	39.5 ± 4.2	38.3 ± 5.4
H group	43.5 ± 6.1	33.6 ± 1.9	32.2 ± 3.9
W group	43.9 ± 5.7	31.5 ± 1.5**	30.7 ± 1.8**

The H group: 6% HAES; the R group: 20mL/kg Ringer's solution; the W group: 6% Voluven. *P < 0.05 compared with baseline value; ^#^*P* < 0.05 compared with the R group.

(%, mean±SD, *n* = 15)

### Changes of heart rates in the three groups during controlled hypotension

The changes of heart rates are displayed in ***Table 3***. No differences were observed in the baseline values of heart rate in the three groups. However, the heart rate was increased gradually in the R group due to the effect of controlled hypotension. There was a significant effect after 1 h (84.5±9.7 *vs* 77.1±8.3, *P* < 0.05) and especially after 2 h (86.4±9.5 *vs* 77.1±8.3, *P* < 0.01). Interestingly, the heart rate gradually decreased after 2 h. When recorded at the third h time point, the changes became similar to the baseline value (*P* > 0.05). Similarly, there was a trend of increase at the first and the second hour time point in both the H group (80.4±9.6, 82.3±8.7 *vs* 76.7±8.5) and the W group (79.5±8.8, 81.8±8.6 *vs* 78.8±8.5), but the effect was insignificant, and no significant difference was observed (*P* > 0.05). Additionally, there was a slight drop in heart rate at the 3 h time point in both two groups. When intergroup difference was analyzed, however, no difference was detected at all the time points during controlled hypotension (*P* > 0.05, ***Table 3***).

**Table 3:** Changes of heart rates of patients in hepatic cancer patients under controlled hypotension and receiving infusion of different fluids

Group	Basic value	Controlled Hypotension (CH)		
		After 1 h	After 2 h	After 3 h
R group	77.1 ± 8.3	84.5 ± 9.7*	86.4 ± 9.5**	82.4 ± 8.3
H group	76.7 ± 8.5	80.4 ± 9.6	82.3 ± 8.7	80.3 ± 8.4
W group	78.8 ± 8.5	79.5 ± 8.8	81.8 ± 8.6	79.2 ± 8.9

bpm: beat per min. the H group: 6% HAES; the R group: 20mL/kg Ringer's solution; the W group: 6% Voluven. Compared with basic value, *P < 0.05, **P < 0.01.

(bpm, mean±SD, *n* = 15)

### Changes of pHa, PaO_2_, PaCO_2_, BE, pHi, and PgCO_2_ before, during and after controlled hypotension

The changes of artery blood gases and gastric mucosal metabolisms are illustrated in ***Table 4***. No significant difference was detected in artery blood gas including pH, PaO_2_ and PaCO_2_ in the three groups at the different time points (*P* > 0.05). Compared with the W group, base excess (BE) was decreased at the 1 h (-2.243±0.522 *vs* 0.921±0.632, *P* < 0.05) and 2 h (-3.306±2.243 *vs* 0.322±0.832, *P* < 0.05) time-point after nitroglycerin-induced controlled hypotension and 1 h (-2.918±0.908 *vs* 0.414±0.721, *P* < 0.05) after the termination of controlled hypotension in the R group, and the BE value in the H group showed no changes at each time point (0.611±0.532, 0.142±1.107 and 0.218±0.921, respectively). However, pHi in the R group was significantly lowered at 1 h (7.391±0.035) and 2 h (7.371±0.015) after the establishment of controlled hypotension compared with that of the W group (7.422±0.025 and 7.418±0.033) (*P* < 0.05). But fortunately, pHi values were still above 7.35. PgCO_2_ [(41.144±4.091) mmHg and (41.972±2.964)mmHg] of the R group was significantly elevated at the same time points compared with that of the W group [(37.872±1.981) mmHg and (39.273±2.151) mmHg] (*P* < 0.05 or *P* < 0.01, ***Table 4***).

**Table 4:** Comparison of artery gases and gastric mucosal metabolisms in hepatic cancer patients under controlled hypotension and receiving infusion of different fluids

Index	Group	basic date	1h after	2h after	1h after termination
pH	R	7.407 ± 0.031	7.402 ± 0.055	7.381 ± 0.048	7.395±0.045
	H	7.410 ± 0.032	7.395±0.053	7.385 ± 0.035	7.402 ± 0.042
	W	7.405 ± 0.032	7.403 ± 0.047	7.395 ± 0.040	7.403 ± 0.038
PaO_2_ (mmHg)	R	435.516 ± 83.534	426.326 ± 78.513	424.927 ± 81.533	419.443 ± 76.619
	H	422.534 ± 79.626	400.336 ± 84.526	398.343 ± 76.527	410.221 ± 58.810
	W	428.725 ± 78.818	408.307 ± 87.443	406.087 ± 87.331	415.108 ± 68.309
PaCO_2_ (mmHg)	R	39.512 ± 2.805	39.834 ± 3.231	41.413 ± 2.421	40.808 ± 2.712
	H	40.109 ± 2.134	41.626 ± 1.843	41.908 ± 2.742	40.842 ± 2.525
	W	41.532 ± 2.841	40.632 ± 1.832	42.521 ± 2.431	41.809 ± 2.236
BE	R	1.522 ± 0.908	-2.243 ± 0.522*	-3.306 ± 2.243*	-2.918 ± 0.908*
	H	1.818 ± 0.707	0.611 ± 0.532	0.142 ± 1.107	0.218 ± 0.921
	W	1.623 ± 0.832	0.921 ± 0.632	0.322 ± 0.832	0.414 ± 0.721
pHi	R	7.425 ± 0.032	7.391 ± 0.035*	7.371 ± 0.015*	7.399 ± 0.013
	H	7.422 ± 0.027	7.401 ± 0.025	7.389 ± 0.023	7.395 ± 0.028
	W	7.418 ± 0.029	7.422 ± 0.025	7.418 ± 0.033	7.416 ± 0.025
PgCO_2_ (mmHg)	R	39.263 ± 1.972	41.144 ± 4.091*	41.972 ± 2.964**	40.233 ± 2.472
	H	39.891 ± 2.113	40.170 ± 2.883	40.820 ± 2.782	39.951 ± 3.212
	W	38.882 ± 2.023	37.872 ± 1.981	39.273 ± 2.151	39.831 ± 2.324

BE: base excess; pHi: intramucosal pH; PgCO_2_: intramucosal PCO_2_; the H group: 6% HAES; the R group: 20 mL/kg Ringer's solution; the W group: 6% Voluven. Compared with the W group, *P < 0.05, **P < 0.01.

(mean±SD, *n* = 15)

### Changes of post-operational gastrointestinal recovery in three groups

Recovery of gastrointestinal function was determined by the first passage of flatus and defecation postoperatively. Time of first passing flatus and defection were recorded by patients, and collected by investigators. Median time to first flatus was (39.5±7.6) h and defecation was (72.6±8.4) h in W group, respectively, which was significantly more rapid comparing with either of the two groups (*P* < 0.05). These two parameters were not significantly different between the R group and H group (*P* > 0.05).

## DISCUSSION

Hypervolemic hemodilution, which can inevitably lead to increased cardiac preload, increases myocardial oxygen consumption and pulmonary capillary leakage, is usually practiced for some patients to save blood[5],[6]. We used hypervolemic hemodilution in the same time to induce hypotension to reduce cardiac stress. Sixty g/L hydroxyethyl starch (HAES200/0.5, HAES; HAES130/0.4, Voluven) is an effective plasma substitute for capacity of the treatment, especially because Voluven has the characteristics of moderate molecular weight, can efficiently expand the plasma volume, and possesses the characteristics of hemorheology that can improve the microcirculation[7]–[10], and prevent capillary leakage[11],[12]. In this study, we expand blood volume using hydroxyethyl starch, and as indicated by the changes in hematocrit before and after volume expansion, we found that hydroxyethyl starch significantly decreased the hematocrit after the infusion, which, though, remained above 0.3. Studies have pointed out that hematocrit between 25% and 30% can offer the maximum amount of oxygen transport, decrease blood viscosity, and improve tissue oxygenation. After compound sodium lactate was infused, more than two thirds of it will move out of blood vessels while only 20% can contribute to capacity expansion and volume expansion is maintained for only 20-30 min.

Controlled hypotension during surgical operation has been widely adopted. More frequently, the protection of gastrointestinal function during controlled hypotension is overlooked, compared with the awareness of surgeons for blood supplies to vital organs such as the heart, brain and kidney[13]–[16]. It is well accepted that the gastrointestinal system is richly perfused and very sensitive to hypoxia and ischemia. Researchers have found that the gastrointestinal mucosa is among the first inflicted organs during tissue ischemia and the last organs that ameliorate after cessation of ischemia[17]–[20]. Reports have shown that exceedingly low pHi value during operation as a result of hypoxia/ischemia of the gastrointestinal mucosa results in slow recovery of gastrointestinal function, breakage of the gastrointestinal barrier and disruption of the epithelial metabolisms. The gastrointestinal mucosa is therefore more permeable to endotoxins and bacterium, which translocate into the blood stream to cause “endogenous” infections[21]–[23], and in severe cases, it can cause even more serious complications such as peptic ulcer. Therefore, monitoring of gastrointestinal pH can reflect not only overall hypoxic conditions of the body, but also the oxygenation states of individual organs.

Due to affluent blood supply of the liver, hepatic surgery is usually more traumatic and blood loss is heavier. In addition, circulation is more prone to fluctuation, resulting in hypotension and arrhythmia[24]. Therefore, controlling blood loss is critical to improve the safety of the operation and anesthesia conditions[25]. In order to control bleeding effectively, we used high-volume hemodilution combined with controlled hypotension[26],[27], resulting in systemic blood volume increase and hematocrit decrease, improving the blood rheology and microcirculation of organ[28],[29]. Studies have confirmed that acute hypervolemic hemodilution with rapid infusion of a large amount of hydroxyethyl starch can produce significant increase in effective circulating blood volume, diluting the circulation of vasoactive substances and the concentration of harmful metabolites, and will be helpful to reduce the stress of the negative impact on organs and tissues. Acute hypervolemic hemodilution can significantly reduce perioperative blood viscosity, hematocrit and erythrocyte aggregation index, and make the tissue blood perfusion improve significantly. In addition, hypervolemic hemodilution also enable red blood cell deformability significantly higher. With incrased deformability, red blood cells can easily transit through the capillaries, thus contributing to tissue oxygen supply. The results of our study also confirmed that pHi of the W group after controlled hypotension was significantly higher than that of the R group. In the H group, pHi seems to be higher than that of the R group, but no significant difference was noted. Perhaps our sample size was too small to render appropriate power, and perhaps Voluven surpasses HAES in fluid expansion and improving microcirculation, pending further study in the future.

pHi could indirectly reflect the supply of oxygenation of tissues and monitor visceral blood flow. The tonometry is the only non-invasive method used in clinic now in evaluating visceral blood flow. By measuring PgCO_2_, the pHi values can be deduced. Therefore, intramucosal acidosis and the decrease of visceral blood flow can be detected at an early stage. It has been shown that an elevated local PgCO_2_ is correlated with poor prognosis in patients undergoing major surgery, suffering severe traumas or being critically ill[30],[31]. The tonometry uses conventional tubes (a type of gastric tube with silicon air bag attached to the end). The air bag is automatically inflated with 5 ml air, which is equilibrated in the gut environment for 10 min before an air sample is measured for CO_2_ pressure using infrared lights. The sampling air is then returned to the air bag, equilibrated, and is then ready for the next measurement. Compared with salt-water tonometry, this approach takes less time to equilibrate, provides excellent accuracy and is more reproducible[32]. This technique can detect reduced gastric mucoal perfusion within a short time period (5 min), and can provide a continuous, automatic monitoring for gut blood perfusion.

In summary, our study has shown that systemic oxygen delivery index (such as PaO_2_) has no significant change in deliberate hypotension with infusion of different liquids, but the BE decreased significantly, gastrointestinal PgCO_2_ increased significantly and pHi was significantly reduced in the R group during controlled hypotension, suggesting a tendency of metabolic acidosis had developed. The results suggested that pHi was a more sensitive index than systemic oxygen delivery in monitoring local tissue oxygen supply. To some extent, it could be used to predict the degree of ischemia and provide us with a reliable and objective basis for early clinical intervention. In addition, pHi, as a non-invasive, simple, reliable, dynamic monitoring method, also could be used in critically-ill patients in the perioperative period.

We believed that infusion of 6% hydroxyethyl starch, especially of Voluven, was beneficial for the microcirculation perfusion. This infusion can also help to save blood and prevent transfusion complications in the perioperative period.
